# Hedgehog/Gli supports androgen signaling in androgen deprived and androgen independent prostate cancer cells

**DOI:** 10.1186/1476-4598-9-89

**Published:** 2010-04-26

**Authors:** Mengqian Chen, Michael A Feuerstein, Elina Levina, Prateek S Baghel, Richard D Carkner, Matthew J Tanner, Michael Shtutman, Francis Vacherot, Stéphane Terry, Alexandre de la Taille, Ralph Buttyan

**Affiliations:** 1 The Ordway Research Institute, Albany, New York, USA; 2 Division of Urology, Albany Medical College, Albany, New York, USA; 3 INSERM U955Eq07 and University of Paris XII, Creteil, France; 4 Department of Pathology and Laboratory Medicine, Weil Cornell Medical College, New York, USA

## Abstract

**Background:**

Castration resistant prostate cancer (CRPC) develops as a consequence of hormone therapies used to deplete androgens in advanced prostate cancer patients. CRPC cells are able to grow in a low androgen environment and this is associated with anomalous activity of their endogenous androgen receptor (AR) despite the low systemic androgen levels in the patients. Therefore, the reactivated tumor cell androgen signaling pathway is thought to provide a target for control of CRPC. Previously, we reported that Hedgehog (Hh) signaling was conditionally activated by androgen deprivation in androgen sensitive prostate cancer cells and here we studied the potential for cross-talk between Hh and androgen signaling activities in androgen deprived and androgen independent (AI) prostate cancer cells.

**Results:**

Treatment of a variety of androgen-deprived or AI prostate cancer cells with the Hh inhibitor, cyclopamine, resulted in dose-dependent modulation of the expression of genes that are regulated by androgen. The effect of cyclopamine on endogenous androgen-regulated gene expression in androgen deprived and AI prostate cancer cells was consistent with the suppressive effects of cyclopamine on the expression of a reporter gene (luciferase) from two different androgen-dependent promoters. Similarly, reduction of smoothened (Smo) expression with siRNA co-suppressed expression of androgen-inducible KLK2 and KLK3 in androgen deprived cells without affecting the expression of androgen receptor (AR) mRNA or protein. Cyclopamine also prevented the outgrowth of AI cells from androgen growth-dependent parental LNCaP cells and suppressed the growth of an overt AI-LNCaP variant whereas supplemental androgen (R1881) restored growth to the AI cells in the presence of cyclopamine. Conversely, overexpression of Gli1 or Gli2 in LNCaP cells enhanced AR-specific gene expression in the absence of androgen. Overexpressed Gli1/Gli2 also enabled parental LNCaP cells to grow in androgen depleted medium. AR protein co-immunoprecipitates with Gli2 protein from transfected 293T cell lysates.

**Conclusions:**

Collectively, our results indicate that Hh/Gli signaling supports androgen signaling and AI growth in prostate cancer cells in a low androgen environment. The finding that Gli2 co-immunoprecipitates with AR protein suggests that an interaction between these proteins might be the basis for Hedgehog/Gli support of androgen signaling under this condition.

## Background

When detected in the advanced stage, prostate cancer patients are treated with hormone therapies that reduce systemic androgen levels [1-3]. This action palliates the symptoms of metastases, induces regression of metastatic lesions and slows prostate tumor growth [4]. Over time, however, the cancer can recur in a castration resistant form (CRPC) that continues to grow despite the ability of hormone therapy to maintain systemic androgens at castrate levels and deaths from prostate cancer are inevitably associated with complications from this form of disease [5]. Progression of prostate cancer to CRPC appears to involve a reactivation of androgen signaling in the cancer cells [6-8] and a variety of mechanisms may account for residual androgen signaling in a low androgen environment. These include expression of variant forms of androgen receptor (AR) that are transcriptionally active without ligand [9,10], acquisition of an ability to endogenously synthesize androgens by the tumor cells themselves [11,12] or activation of aberrant AR transcriptional activity through cross-talk with alternate signaling pathways [6,13]. While all of these mechanisms are of interest from a scientific viewpoint, the ones that are readily targetable by drugs are the most clinically imperative as they offer an opportunity to test novel therapies to treat a disease that will kill almost 28,000 men in the United States this year. Recent reports that Abiraterone, an inhibitor of androgen biosynthesis, has clinical effects against castration resistant prostate cancer, reflects a potential treatment advance that might target tumor cell androgen biosynthesis [14]. Here we describe findings that suggest that inhibitors of the Hedgehog/Gli signaling pathway, currently in clinical testing for a variety of cancers, might also have a role for the treatment of castration resistant prostate cancer due to an ability to suppress reactivated androgen signaling in tumor cells.

Hedgehog (Hh) is best known for its role in tissue patterning and morphogenesis during embryonic development [15-18]. In the developmental situation, Hh is a ligand-driven process in which a ligand (referred to as a Hedgehog) engages the Patched 1 (Ptch) receptor on the cell surface and this relieves repression of Smoothened (Smo), a member of the extended G protein coupled receptor family [18]. Smo, when activated, then acts downstream to alter the processing and intracellular localization of Gli transcription factors and to increase Gli-mediated transcriptional activity. The plant-derived alkaloid, cyclopamine, is a prototype for a drug that antagonizes the Hh signaling process [19]. Cyclopamine antagonizes Smo activation and this action explains the teratogenic effects of this drug when it is ingested during pregnancy [20,21].

Aside from its role in development, Hh signaling also supports stem cells in adult tissues [22-24]. However, chronically hyperactive Hh/Gli signaling in adult tissues can be oncogenic, especially for the skin or brain [25,26]. Basal cell carcinoma of the skin and medulloblastoma are models for human Hh-mediated oncogenesis [27]. The aberrant Hh activity in these tumors can result from a loss of the Ptch gene or its function [28,29], mutations in Smo [30] or SuFu [31] that activate endogenous Hh signaling or cryptic overexpression of Gli proteins in tumor cells. For prostate cancer, the question as to whether Hh/Gli signaling plays any role is controversial. Although cyclopamine treatment or Gli knockdown suppresses the *in vitro *growth of prostate cancer cell lines or xenograft tumor growth in mice [32-34], the commonly used prostate cancer cell lines show little, if any, evidence for active canonical Hh signaling activity when they are grown in standard culture conditions [35,36]. For the androgen-growth dependent LNCaP prostate cancer cells and its variants, C4-2 and C4-2B, however, the situation was found to be changed by chronic exposure of these cells to androgen depleted medium. Androgen deprivation highly upregulated the expression and secretion of Hh ligands and increased endogenous expression of Hh/Gli target genes in these cells [37]. The clinical relevance of this observation is supported by the observation that Hh ligand production was found to be increased in prostate tumors by neoadjuvant hormone treatment [38]. Since cyclopamine suppresses the expression of Hh target genes in androgen-deprived LNCaP cells (37), this also suggests that active Hh/Gli signaling activity is awakened by growth under androgen deprived conditions. Others have observed that the high basal expression of Hh/Gli target genes in androgen independent (AI) variants of LNCaP was reduced by cyclopamine [39] and, collectively, the outcomes of these studies imply that Hh signaling in LNCaP cells is restricted to the androgen deprived or AI state. The question remains as to whether active Hh signaling has any biological consequences for the androgen deprived or AI prostate cancer cell. Here we show that, by manipulating the activity of canonical Hh signaling in androgen deprived or AI prostate cancer cells, we also affected the expression of androgen regulated genes and the ability of these cells to grow in the absence of androgen. Our results indicate that Hh/Gli signaling activity supports androgen signaling and AI growth in prostate cancer under low/no androgen conditions. Furthermore, we report that Gli2 protein can bind to AR and this interaction might define the point of cross-talk between the two signaling pathways.

## Results and Discussion

**Figure 1:**
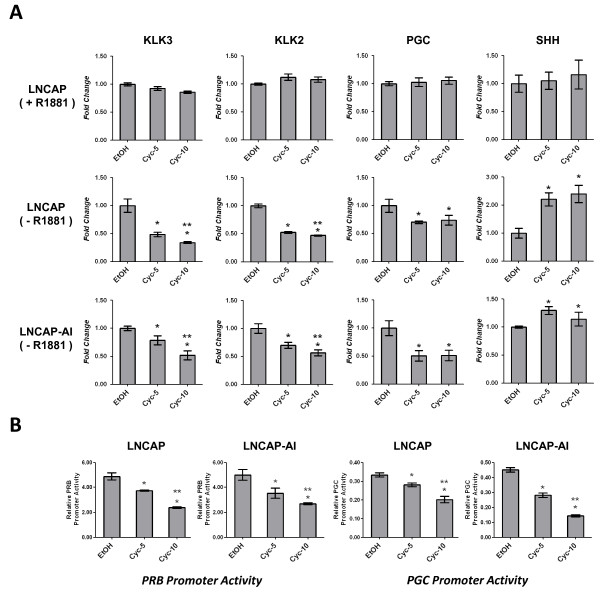
**Effect of cyclopamine on androgen signaling in LNCaP cells**. (A) Real time qPCR was used to measure relative expression of KLK3, KLK2, PGC or SHH mRNA in androgen-supplemented (+R1881) or androgen deprived (-R1881) LNCaP or in LNCaP-AI cells (-R1881) in the presence of vehicle (EtOH) or with 5 or 10 μM cyclopamine (Cyc-5, Cyc-10) (also see Additional file 2, Table S2). (B) LNCaP or LNCaP-AI cells were infected with probasin (PRB) or PGC promoter reporter vectors along with a CMV-GFP reference reporter and were cultured in androgen depleted medium with vehicle (EtOH) or with 5 or 10 μM cyclopamine (Cyc-5 or Cyc-10) for 72 hrs. Cell extracts were assayed for luciferase that was normalized by GFP intensity. Bars represent the means of triplicate experiments ± S.E. (* = P < 0.05 compared to vehicle control; ** = P < 0.05 between 5 and 10 μM cyclopamine treatment groups).

Previously we reported evidence for conditional activation of canonical Hh signaling in androgen sensitive human prostate cancer cells by culture in an androgen depleted conditions [37]. Here, we used androgen sensitive parental LNCaP cells, other derivatives of LNCaP that are less dependent on androgens for growth (C4-2, LN3, LNCaP-AI) or androgen responsive VCaP cells that are unrelated to LNCaP, to study the effects of Hh signaling manipulation on the expression of androgen regulated genes in these cells. The LNCaP-AI variant cells that we used were independently isolated in our lab following long-term (> 1 year) culture of parental LNCaP cells in androgen depleted medium. These cells downregulate basal expression of Ptch1 when treated with cyclopamine (Additional file 1, Figure S1) so they appear to have basal-active Hh signaling activity similar to other AI derivatives of LNCaP that were previously described (39). Initially, we tested the effects of the classic Hh inhibitor drug, cyclopamine on androgen regulated gene expression. All experiments were done using a medium that was depleted for androgens (phenol red-free RPMI with charcoal-stripped FBS) that could be re-supplemented with androgen (R1881) to mimic androgen-stimulated conditions. For parental LNCaP cells grown in androgen supplemented medium (+R1881), the presence of cyclopamine had no significant effects on the expression of four model androgen-regulated genes; KLK2, KLK3 [PSA] and PGC (androgen-inducible), or SHH that is repressed by androgen (Figure 1A). However, when these cells were switched to androgen depleted medium (-R1881) for 3 days, cyclopamine treatment had a distinct dose-dependent effect that further suppressed expression of KLK2, KLK3 and PGC and further increased expression of SHH (Figure 1A). Likewise, cyclopamine significantly downregulated expression of KLK2, KLK3 and PGC in the LNCaP-AI cells that are normally propagated in androgen-free medium, and it upregulated the expression of SHH in these cells (Figure 1A). Cyclopamine also suppressed the expression of KLK2 and KLK3 in VCaP, LN3 or C4-2B cells grown in androgen depleted medium for 3 days (Additional file 1, Figure S2), so the effects of cyclopamine on androgen regulated genes were not limited to LNCaP or its derivatives. We also tested whether a more water-soluble cyclopamine derivative, KAAD-cyclopamine, had a similar effect and found that this drug (at 0.5 or 1 μM) was as effective in reducing KLK2/3 and PGC expression in androgen-deprived LNCaP or LNCaP-AI cells as the 5 or 10 μM dose of cyclopamine (Additional file 1, Figure S3). Finally, we found that cyclopamine also significantly diminished the expression of a reporter gene (luciferase) from either of two androgen dependent promoters (Probasin [PRB] or PGC) in LNCaP or LNCaP AI cells in androgen depleted medium (Figure 1B) in a dose dependent manner. As for endogenous androgen-regulated genes, cyclopamine did not affect the expression of the reporter when cells were cultured in medium supplemented with 10 pM R1881 (data not shown).

**Figure 2:**
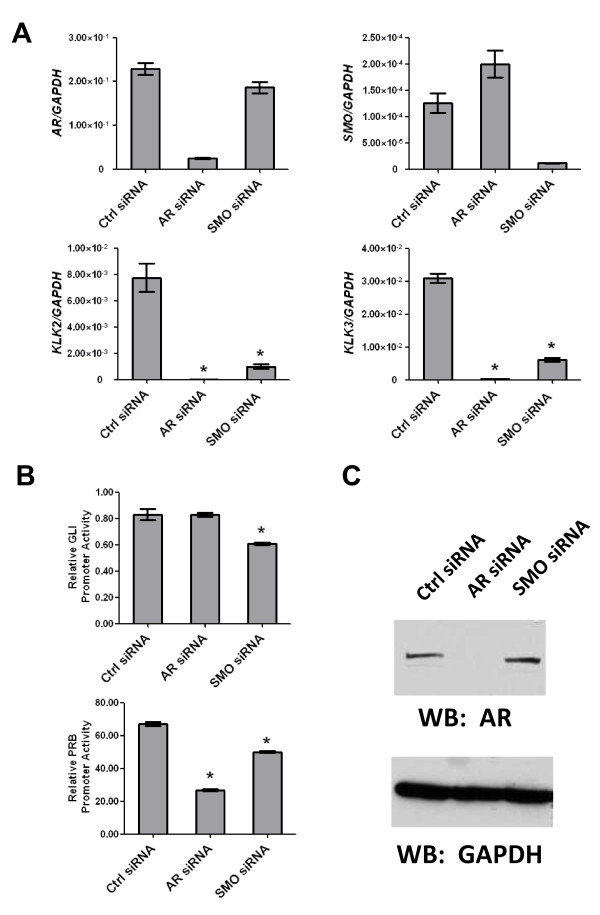
**Smo knockdown affects androgen responsive gene expression in androgen-deprived LNCaP cells**. (A) LNCaP cells were transfected with control (Ctrl) siRNA, AR or Smo siRNA and grown in androgen-depleted medium for 72 hrs. RNAs were extracted and assayed by real-time qPCR for expression of AR, Smo, KLK2 or KLK3. Bars represent the means of three experiments ± S.E. (* = P < 0.05 compared to control siRNA). (B) Cells transfected with siRNA were infected with a Gli or Probasin (PRB) FF luciferase reporter lentivirus along with a CMV-GFP lentivirus control reporter and were switched to androgen-depleted medium for 72 hrs. Cell extracts were quantified for luciferase activity that was normalized by GFP intensity. Bars represent the means of triplicate experiments ± S.E. (* = P < 0.05 compared to control siRNA). (C) Western blot shows effects of siRNA on expression of AR protein in cell lysates.

Cyclopamine represses Hh signaling through its ability to antagonize Smo activation so we also tested whether Smo expression knockdown (using siRNA) could mimic the effects of cyclopamine with regards to suppression of androgen-inducible gene expression. LNCaP cells were transfected either with control (non-targeting) siRNA or with siRNA targeting AR or Smo and were thereafter maintained in androgen-depleted medium. AR siRNA selectively reduced expression of AR mRNA and protein (Figures 2A, C) but did not reduce the expression of Smo. Likewise, Smo siRNA reduced Smo mRNA levels but did not affect expression of AR mRNA or protein (Figure 2C). However, both AR and Smo siRNAs similarly reduced expression of KLK2 and KLK3 (Figure 2A). Further assessment of the effects of AR or Smo siRNA on expression of a luciferase reporter from either a Gli- or androgen-responsive promoter showed that AR knockdown selectively reduced expression of the androgen reporter but did not affect expression of the Gli reporter (Figure 2B). In contrast, Smo knockdown significantly reduced expression of both the Gli and androgen reporters (Figure 2B) in androgen deprived LNCaP cells. In summary, the above data shows that suppression of Hh signaling with a Smo antagonist, cyclopamine, or by reduction of Smo expression itself, suppresses expression of androgen inducible genes and induces expression of androgen repressed genes, but only when these human prostate cancer cells were cultured in a medium lacking androgen. The fact that Smo knockdown reduced expression of androgen regulated genes but did not affect expression of AR mRNA or protein suggests that some aspect of Hh signaling regulates the activity of the AR rather than its expression.

**Figure 3:**
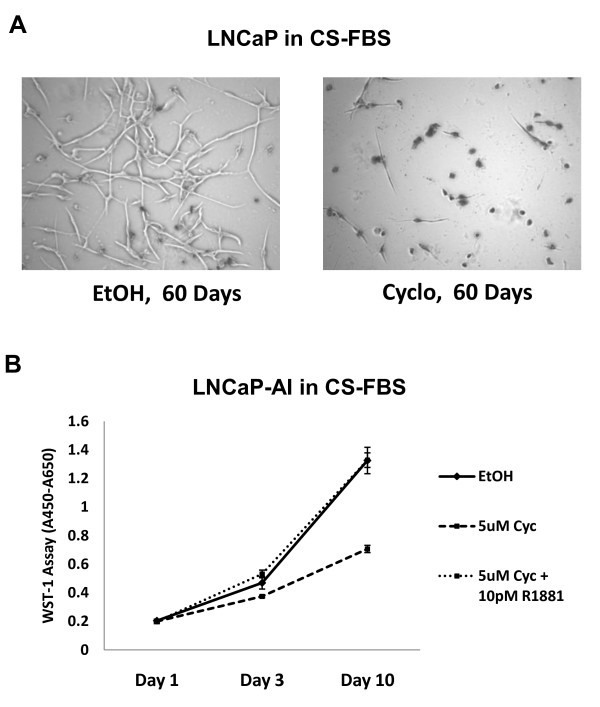
**Cyclopamine prevents the development of AI prostate cancer cell growth and suppresses the growth of LNCaP-AI cells**. (A) Phase contrast photomicrographs (40×) of LNCaP cells cultured for 60 days in androgen depleted medium (CS-FBS) supplemented with vehicle (EtOH) or 5 μM cyclopamine (Cyclo). Cell numbers in cyclopamine are greatly reduced and cells have optically dense, fragmented nuclei. (B) LNCaP-AI cells grown in androgen-depleted medium (CS-FBS) supplemented with vehicle (EtOH) or 5 μM cyclopamine. Cell numbers were counted at various days as indicated. Points represent the means of triplicate cultures ± S.E.

Since cyclopamine suppressed residual/reactivated androgen gene expression in androgen deprived and AI prostate cancer cells, we also sought evidence that this effect had biological consequences relevant to AI growth. First, we tested whether the presence of cyclopamine might prevent the development of AI cells from parental LNCaP cells chronically maintained in androgen depleted medium. LNCaP cells were seeded onto 10 plates at low density and then 5 plates each were switched to androgen depleted medium supplemented with vehicle (EtOH) or with 5 μM cyclopamine. The media were changed every 3 days. Within 2 months, cell numbers in the cyclopamine-treated cultures were significantly reduced compared to vehicle-treated cultures and most surviving cells in the cyclopamine-treated cultures were shrunken with optically dense nuclei that contrasted with the neuroendocrine cell-like appearance of cells in vehicle-treated cultures (Figure 3A). By the third month, cyclopamine-treated cultures had less than 1% of the cells of vehicle-treated cultures and all remaining cells showed the presence of the optically dense nuclei. No cells remained on cyclopamine-treated plates by 4 months of culture but the cells in the vehicle-treated cultures were increasing in numbers by this time and these cultures gave rise to growing lawns of cells by 6 months that typify AI growth. For overt LNCaP-AI cells, we found that treatment with 5 μM cyclopamine significantly inhibited their growth over a 10 day period (Figure 3B) but when cyclopamine treatment was accompanied by supplemental androgen (10 pM R1881), the growth rate of these cells was no different than vehicle treated cells. This indicates that the presence of androgen can overcome the growth-inhibiting effects of cyclopamine on overt AI cells.

**Figure 4:**
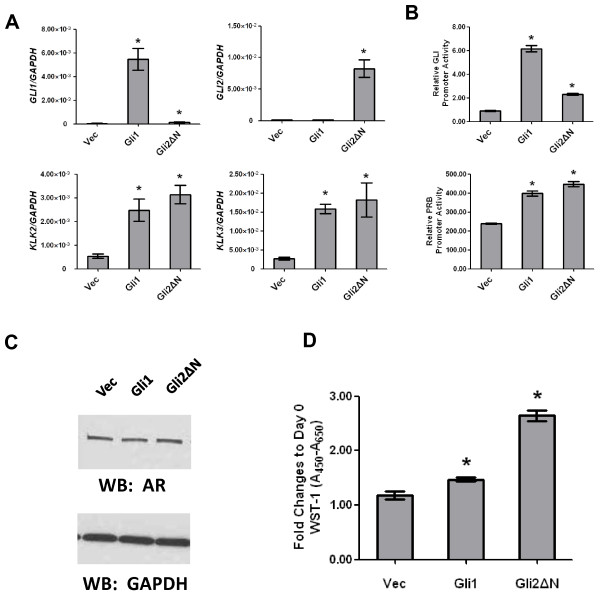
**Gli overexpression affects androgen regulated gene expression in androgen-deprived LNCaP cells**. (A) RNAs from control (Vec) or Gli1 or Gli2 (Gli2ΔN) overexpressing LNCaP cells cultured in androgen-depleted medium for 72 hrs were assayed by real-time qPCR for expression of Gli1, Gli2, KLK2 and KLK3. Bars represent the means of three experiments ± S.E. (* = P < 0.05 compared to vector control). (B) Cells were infected with a Gli or Probasin (PRB) reporter with CMV-GFP and switched to androgen-depleted medium for 72 hrs. Cell extracts were quantified for luciferase that was normalized by GFP intensity. Bars represent the means of triplicate experiments ± S.E. (* = P < 0.05 compared to vector control). (C) Western blot shows that Gli1 or Gli2 overexpression does not affect expression of AR protein. (D) Gli overexpression enables androgen independent cell growth. Control (Vec) or Gli1 or Gli2 (Gli2ΔN) overexpressing LNCaP cells were cultured in androgen depleted medium for 12 days and growth was measured by WST-1 assay and compared to Day 0. Bars represent the means of three experiments ± S.E. (* = P < 0.05 compared to vector control).

Finally, we sought to test whether overexpression of Gli1 or Gli2, transcription factors that lie at the endpoint of the Hh signaling process, might act oppositely to Smo antagonism/inhibition to increase androgen signaling or AI growth when LNCaP cells were grown in androgen free medium. Parental LNCaP cells were transduced with a Gli1- or Gli2- (Gli2ΔN) expressing lentivirus and these cells were compared to control cells transduced with empty virus to determine the effects of Gli overexpression on androgen regulated gene expression and cell growth in androgen depleted medium. The Gli overexpressing variants of LNCaP were also found to express significantly higher levels of KLK2 or KLK3 when compared to control (vector transduced) cells in androgen depleted medium (Figure 4A). Gli1 or Gli2 overexpressing LNCaP cells also expressed significantly higher levels of luciferase reporter from both AR and Gli dependent promoters compared to control cells (Figure 4B). Despite higher basal expression of androgen regulated genes, the Gli transduced cells expressed AR protein at equivalent levels to the control cells (Figure 4C) so here again, these effects appear to be independent of changes in AR expression. The Gli transduced LNCaP cells also showed significant increased growth in androgen depleted medium compared to the control cells (Figure 4D), though Gli2 cells appeared to be more robust than Gli1 in this regard. Regardless of this differential hierarchy, this data shows that Gli function supports androgen regulated gene expression in a low androgen environment as well as AI growth.

**Figure 5:**
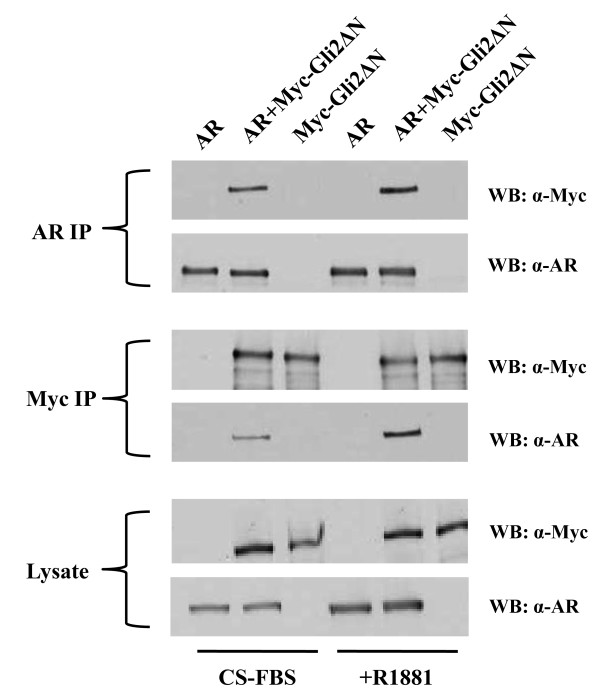
**Co-immunoprecipitation of AR with Gli2 protein**. Lysates of cells (293FT) transfected with AR, myc-tagged Gli2 (Myc-Gli2ΔN) or both for 48 hrs (under androgen-supplemented [R1881] or depleted [CS-FBS] conditions) were immunoprecipitated with α-AR or α-myc antibody. IPs or lysates were electrophoresed and blotted. The Western blot (WB) was probed with α-AR or α-myc antibody as indicated.

The evidence that Gli1 or Gli2 overexpression upregulates androgen inducible gene expression and AI growth of androgen deprived LNCaP cells without affecting AR expression suggests that some function of the Gli proteins may support AR transcriptional activity in a low androgen environment. We tested for some potential direct interaction between these Gli and AR proteins in co-immunoprecipitation experiments. Human 293FT cells were transfected with an expression plasmid for full-length human AR, myc-tagged Gli2 or a combination of these plasmids. Forty-eight hrs later, extracts from the cells were immunoprecipitated with anti-AR or anti-myc antibody and the immunoprecipitates (IPs) were analyzed by Western blot for the presence of AR or myc-tagged Gli2. When the Western blot was probed with anti-AR, we found that AR co-immunoprecipitated with myc-tagged Gli2 only in extracts from cells co-transfected with both plasmids (Figure 5). Similarly, myc-tagged Gli2 was co-immunoprecipitated in the AR IPs from extracts of cells co-transfected with both plasmids (Figure 5). This apparent interaction between Gli2 and AR in the 293FT cells was not diminished by supplementation with 1 nM R1881.

Here we provided evidence that aspects of the canonical Hh signaling pathway can play a role in supporting residual/reactivated androgen signaling in androgen deprived and AI prostate cancer cells and this finding has important implications with regards to both the mechanistic basis for AI growth in the castration resistant prostate cell and for treatment strategies for CRPC in patients. Smo inactivation by cyclopamine, a cyclopamine variant drug (KAAD-cyclopamine) or reduction in Smo expression by siRNA downregulated androgen inducible genes in androgen deprived and AI prostate cancer cells and these findings suggest that some action of Smo might be important for reactivation of androgen signaling under low androgen conditions. The effects of cyclopamine on androgen regulated genes was common to several types of human prostate cancer cell lines grown under androgen deprived conditions so the effect was not limited to LNCaP cells and derivatives. Cyclopamine also suppressed expression of reporter genes from two different androgen responsive promoters in LNCaP cells in androgen depleted medium and these findings support the idea that Smo activity supports AR-mediated transcriptional activity in the androgen deprived state. Finally, the modulatory effects of cyclopamine on AR regulated gene expression were consistent with the effect of this drug on AI growth. Chronic cyclopamine treatment prevented the development of androgen growth independent cells from parental androgen growth-dependent LNCaP cells and significantly inhibited the growth of an overt AI variant of LNCaP. The cyclopamine-mediated growth suppression was reversed by returning a low level of androgen to the cells, providing further evidence that effects of cyclopamine on development and growth of AI cells are based upon cyclopamines' actions on residual androgen signaling.

Smo action ultimately drives transcription by Gli family proteins so we also tested whether exogenous expression of active Gli had opposite effects of cyclopamine or Smo reduction. Here, our findings that Gli1 or Gli2 overexpression enhanced androgen regulated gene expression in androgen depleted medium and enabled AI growth for androgen growth-dependent cells strongly argues that the active Gli proteins resulting from Hh signaling play the most critical role in Hh-support of residual/reactivated androgen signaling regulation. Although the Gli2 overexpressing LNCaP cells exhibited more robust androgen independent growth than the Gli1 overexpressing cells, it is not possible to rank the effectiveness of the Gli proteins on growth control from this study since the cells may be expressing different amounts of transcriptionally active Gli protein. However, the recent report that Gli2 protein was abundantly expressed in tumor cells from patients with AI (CRPC) prostate cancer [40] does provide further support for the idea that Gli2 protein expression might have a specific role in AI cancer cell growth in CRPC patients and Gli2 may be the preferred target for CRPC treatments.

With regards to the potential mechanism(s) through which Hh/Gli cross-talks to the androgen signaling pathway, it does not appear to involve changes in the expression of AR mRNA or protein as this was not affected by cyclopamine, Smo knockdown or Gli overexpression. However, the finding that Gli2 and AR proteins co-immunoprecipitate when they were co-expressed in 293T cells does suggest that Gli2 might directly interact with AR to influence the expression of AR target genes in the same manner that other co-activator proteins support AR function [41]. Previously Gli2 was shown capable of binding to CREB or to Zic family transcription factor proteins [42,43] so this finding extends the potential repertoire of transcription factors capable of interacting with Gli2. It is of further interest that the interaction between AR and Gli2 proteins was not diminished by androgen supplementation. Therefore, the lack of effects of cyclopamine on androgen regulated gene expression in androgen supplemented LNCaP cells might be due to some additional role of other upstream elements of the Hh signaling pathway that are only manifest in androgen depleted cells. Additionally, we must consider the possibility that Hh/Gli signaling is involved in the endogenous production of androgen (intracrine androgen biosynthesis) that is reportedly associated with AI prostate cancer cells [11], especially since Hh signaling is required for steroidogensis in the testis and for androgen production by other types of cells [44,45]. This is an aspect that we will test for in future experiments.

Regardless of the mechanism(s) involved, the outcome of this research suggests that Hh/Gli inhibitors offer a specific means to target reactivated androgen signaling in CRPC and to test the idea that inhibition of anomalous androgen signaling in CRPC cells has therapeutic benefit for patients. Although cyclopamine is difficult to use as a therapeutic agent, several pharmaceutical companies are in the process of developing similar drugs that are easier to use in the clinical setting and some of these drugs are through Phase I testing [46]. Therefore, translation of these experimental studies to patients should be able to proceed fairly rapidly. Alternatively, there are non- canonical signaling pathways that increase Gli activity in cancer cells [47] so a clinical focus on Smo antagonists may not be sufficient to deal with all forms of CRPC. Reports of small molecular inhibitors of Hh/Gli signaling that act independently of Smo antagonism [48], suggests that Hh/Gli signaling provides a rich array of targets for the development of more effective treatments for CRPC.

## Conclusions

Modulation of Hh signaling in prostate cancer cells by reduction of Smo expression or activity or by overexpression of active Gli proteins affected androgen signaling and the expression of androgen regulated genes in these cells but only when they were cultured in a low androgen medium. The effects of Hh modulation on androgen regulated gene expression in prostate cancer cells were consistent with the coordinate effects on AI cancer cell development and growth in low androgen medium but these effects were reversed by the presence of androgens. Since we have found that Gli2 protein, at least, interacts with the AR protein, the mechanism through which Hh signaling affects AR-dependent gene expression and AI cell growth may involve a direct interaction of AR with Gli proteins.

## Methods

### Cells and Culture

Human prostate carcinoma cell lines LNCaP and VCaP were obtained from the ATCC (Manassas, VA). LNCaP variants, LN3 or C4-2B were obtained from Curtis Pettaway, M.D. Anderson Cancer Center (Houston, TX) or ViroMed Laboratories (Minnetonka, MN), respectively. The LNCaP-AI variant was derived from parental LNCaP cells after more than one year growth in androgen-depleted medium. Cells were maintained in RPMI-1640 medium with 10% fetal bovine serum (FBS) or switched to phenol red-free RPMI-1640 with 10% charcoal-stripped FBS (CS-FBS) for androgen-depleted conditions as previously described (37). The 293FT cells were obtained from Invitrogen, Inc. (Carlsbad, CA) and were maintained in DMEM with 10% FBS. Synthetic androgen, R1881 (methyltrienolone), was obtained from PerkinElmer Life Sciences (Boston, MA) and was supplemented to androgen-depleted medium at 10 pM where indicated. Cyclopamine was obtained from Enzo Life Sciences, Intl. (Plymouth Meeting, PA) and KAAD-cyclopamine from Toronto Research Chemicals Inc. (North York, ON, Canada). Cultured cells were imaged by a Leica DMIRE2 inverted microscope (Leica Microsystems Inc., Bannockburn, IL).

### Generation of LNCaP Lines Stably Expressing Gli Transcription Factors

The ViraPower™ Lentiviral Expression System (Invitrogen) was used for generating replication-incompetent lentiviruses expressing recombinant human Gli1 or Gli2ΔN. All procedures were performed according to the manufacturers' protocols with modifications: 1) cDNAs encoding the full-length human Gli1 and the N-terminal-truncated human Gli2 were cloned from the plasmid GLI K12 [49] and pCS2-MT GLI2(ΔN) [50] (Addgene, Cambridge, MA) into pLenti6 (Invitrogen); 2) For production of lentivirus in 293FT cells, 3 μg of pLenti6-Gli1, pLenti6-Gli2ΔN or pLenti6-Vec (empty vector control) were mixed with 9 μg of ViraPower Packaging Mix, and 36 μl of Lipofectamine-2000 (Invitrogen). The mixture was applied to 2 × 10^6 ^293FT cells in medium overnight. Transfection medium was removed and fresh medium was added for another 72 hours. Lentivirus containing medium was collected and filtered and used for infections; 3) LNCaP cells were seeded at 50% confluence overnight in preparation for viral transduction. Virus supernatants were added (diluted 1:5 with medium) and 48 hrs later, blasticidin was added at a concentration of 10 μg/ml for selection. Selection was carried out for 2-3 weeks and ~200 colonies were obtained and pooled as stably-expressing sublines, LNCaP-Vec, LNCaP-Gli1, or LNCaP-Gli2ΔN.

### RNA Isolation and Reverse Transcription - Real-Time PCR Assays (RT-qPCR)

RNA was isolated from cells using the RNeasy Mini Kit with RNase-Free DNase digestion (QIAGEN, Valencia, CA). Reverse transcription was carried out using SuperScript^® ^III First-Strand Synthesis SuperMix for qRT-PCR (Invitrogen) per the supplier's protocol. Real-time PCR was performed on an ABI 7900HT detection system (Applied Biosystems, Foster City, CA) using RT^2 ^SYBR Green/ROX qPCR Master Mix (SABiosciences, Frederick, MD) according to the manufacturer's protocol. The thermal cycling conditions were as previously described (37). The message number of glyceraldehyde-3-phosphate dehydrogenase (GAPDH) was used as the reference for calculating specific gene messages. The sequences of qPCR primers used are listed in Additional file 2, Table S1.

### Promoter activity assays

Firefly luciferase reporter vectors under the control of a promoter containing eight repeats of the Gli consensus sequence (pLLRM-GLI-Luc) was generated by sub-cloning the GLI-responsive promoter fragment from pGL3B/8XGliBS-lc-luc (JHU-73, ATCC) into a lentiviral luciferase reporter vector, pLLRM. Reporter vectors with rat probasin (PRB) or human Pepsinogen C (PGC) gene promoters and a reference construct expressing GFP under the CMV promoter (pLLCM-GFP) were prepared (Ohouo *et al*., in preparation) and were used to produce lentiviruses in 293FT cells as described above. Cells were lysed 72 hrs after infection with Passive Lysis Buffer (Promega, Madison, WI) and lysates were analyzed for luciferase activity with the 20/20 n Single Tube Luminometer (Turner Biosystems Inc., Sunnyvale CA) using a Luciferase Assay Kit (Promega). GFP intensity was measured by the BMG FLUOstar Optima plate reader (Imgen Technologies, Alexandria, VA) and used to normalize viral-infection efficiency.

### Silencing AR and Smo expression in LNCaP cells by siRNA transfection

The siRNAs specifically targeting human Smo, human AR and control siRNA were purchased from QIAGEN. LNCaP cells were seeded at 70% confluence. siRNAs (40 pM) were mixed with 3 μl of SiLentFect Lipid Reagent for RNAi (Bio-Rad, Hercules, CA) in Opti-MEM I (Invitrogen) for 20 min and this was added to each well in 1.5 ml of medium. Medium was changed 24 hrs after transfection and 72 hrs later, cells were collected for total RNA isolation or lysed in RIPA buffer for Western blot analysis.

### Western blot analysis

Cells lysates were assayed for protein and equal amounts of protein were analyzed by Western blot with appropriate antibodies. Each membrane was re-blotted with GAPDH antibody as a control for protein loading. Antibodies were used at the following dilutions: GAPDH at 1:5,000, AR at 1:10,000, and Myc at 1:5,000. Appropriate secondary antibodies conjugated to horseradish peroxidase were used at 1:10,000, and blots were developed by enhanced chemilluminescence reagent (Thermo Fisher Scientific Inc., Rockford, IL). Antibodies to GAPDH or AR receptor (H-280) were from Santa Cruz Biotechnology, Inc. (Santa Cruz, CA). The monoclonal antibody to Myc-tag (4A6) was purchased from Millipore (Billerica, MA).

### Cell Proliferation WST-1 Assay

Cells were seeded onto a 96-well plate at a density of 5,000 cells/well in CS-FBS media and were maintained for indicated days (media refreshed every 3 days). At appropriate times, 10 μl WST-1 (Roche, Indianapolis, IN) was added to each well and plates were kept at 37°C for two hrs. Color intensity was read at 450 nm (reference wavelength 650 nm) on the SpectraMax M2 microplate reader (Molecular Devices, Sunnyvale, CA)

### Co-immunoprecipitation of AR and Gli2 in 293FT cells

Transfection of 293FT cells (2 × 10^6 ^cells) with AR or Gli2ΔN plasmids was carried out with Lipofectamine-2000. Cells were lysed in a 1% Triton X-100 lysis buffer with protease inhibitor cocktail (Roche) 48 hrs later. Aliquots of extract containing equal amounts of protein were precipitated at 4°C overnight with 50 μl Dynabeads Protein G (Invitrogen) pre-bound with 5 μg appropriate antibodies. Beads were washed by lysis buffer four times and immunoprecipitated proteins were eluted in 2× SDS sample buffer. The elutant was split into equivalent portions and blotted onto 2 membranes for Western blot analysis.

### Statistical Analysis

Expression levels determined using RT-qPCR and promoter activity assay data were compared by comparison of the "means", wherein the data graphed or listed in the table represent the Means ± Standard Error (S.E.). The *Student t-Test *(one-tailed, equal variance) was employed for assessing statistical difference (defined as when p < 0.05) between data groups.

## List of Abbreviations Used

AI: Androgen Independent (Growth); AR: Androgen Receptor; CRPC: Castration Resistant Prostate Cancer; Cyc: Cyclopamine; EtOH: Ethanol; GAPDH: Glyceraldehyde-3-Phosphate Dehydrogenase; Hh: Hedgehog; KLK2: Kallikreinin 2; KLK3: Kallikreinin 3 (Prostate Specific Antigen); IP: Immunoprecipitate; PRB: Probasin; PGC: Pepsinogen C; PSA: Prostate Specific Antigen; Ptch: Patched 1; SHH: Sonic Hedgehog; Smo: Smoothened; Vec: Vector;

## Competing interests

The authors declare that they have no competing interests.

## Authors' contributions

MC conducted the majority of bench experimentation involved in this manuscript and assisted in experimental design and manuscript drafting and editing. MAF conducted some qPCR bench experimentation and reviewed the manuscript for accuracy. EL conducted promoter activity assays and reviewed the manuscript for accuracy. RDC conducted some qPCR bench experimentation and reviewed the manuscript for accuracy. MJT prepared some vectors used in the experimentation and reviewed the manuscript for accuracy. MS prepared some vectors used in the experimentation and reviewed the manuscript for accuracy. FV conducted confirmatory experimentation using the same cells in his laboratory and reviewed the manuscript for accuracy. ST conducted confirmatory experimentation using the same cells in the Vacherot laboratory and reviewed the manuscript for accuracy. AdlT provided funding for the confirmatory experimentation in France and reviewed the manuscript for accuracy. RB provided funding for the experimentation in the US, was responsible for experimental design and data oversight and review and drafted and edited the final manuscript. All authors read and approved the final manuscript.

## Supplementary Material

Additional file 1 Supplemental Figures S1-S3 and the Legends for the Figures. Click here for file

Additional file 2 **Supplemental Tables S1-S2; List of PCR primer sets used in experiments and real-time data for Figure**1. Click here for file
