# Supplementary material for: Hedgehog/Gli supports androgen signaling in androgen deprived and androgen independent prostate cancer cells
Source: Mol Cancer. 2010 Apr 26;9:89. doi: 10.1186/1476-4598-9-89 (PMC2873440; doi:10.1186/1476-4598-9-89)
Supplement: Additional file 1 — Supplemental Figures S1-S3 and the Legends for the Figures. [file 1476-4598-9-89-S1.PDF]

Figure S1. ***Effect of cyclopamine on expression of Ptch1 in LNCaP-AI cells.*** LNCaP-AI cells were cultured in the presence of vehicle (EtOH) or in the presence of 5 or 10 μM cyclopamine (Cyc-5 or Cyc-10) for 3 days and extracted for qPCR analysis of Ptch1 mRNA expression. Bars represent the mean of triplicate experiments ± S.E. (*=P<0.05 compared to vehicle control).

Figure S2. ***Effects of cyclopamine on expression of KLK2 or KLK3 in androgen-deprived VCaP, LN3 or C4-2B cells.*** Real time qPCR was used to measure expression of KLK2 or KLK3 (PSA) in androgen-deprived VCaP, LN3 or C42B cells grown in the presence of vehicle (EtOH) or 5 or 10μM cyclopamine (Cyc-5 or Cyc-10) for 72 hrs. Bars represent the mean of triplicate experiments ± S.E. (*=P<0.05 compared to vehicle control; **=P<0.05 between 5 and 10 μM cyclopamine treatment groups).

Figure S3. ***Effect of KAAD-cyclopamine on KLK2, KLK3, PGC in LNCaP or LNCaP-AI cells.*** Real time qPCR was used to measure expression of KLK2, KLK3 (PSA), or PGC in androgen-deprived LNCaP or in LNCaP-AI cells in the presence of vehicle (EtOH) or 0.5μM (For LNCaP) or 1μM (For LNCaP-AI) KAAD-cyclopamine (KAAD-Cyc) for 72 hrs. Bars represent the means of triplicate experiments ± S.E. (*=P<0.05 compared to vehicle control).

Figure S1

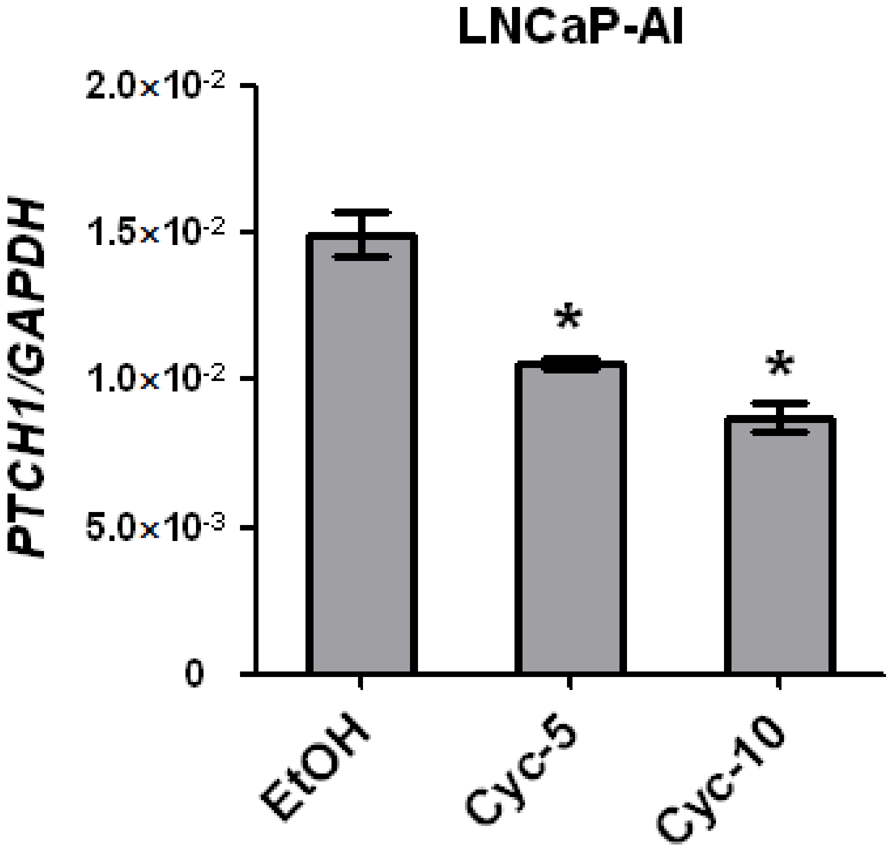
LNCaP-AI
PTCH1/GAPDH
2.0×10-2
1.5×10-2
1.0×10-2
5.0×10-3
0
*
*
EtOH
Cyc-5
Cyc-10

Figure S2

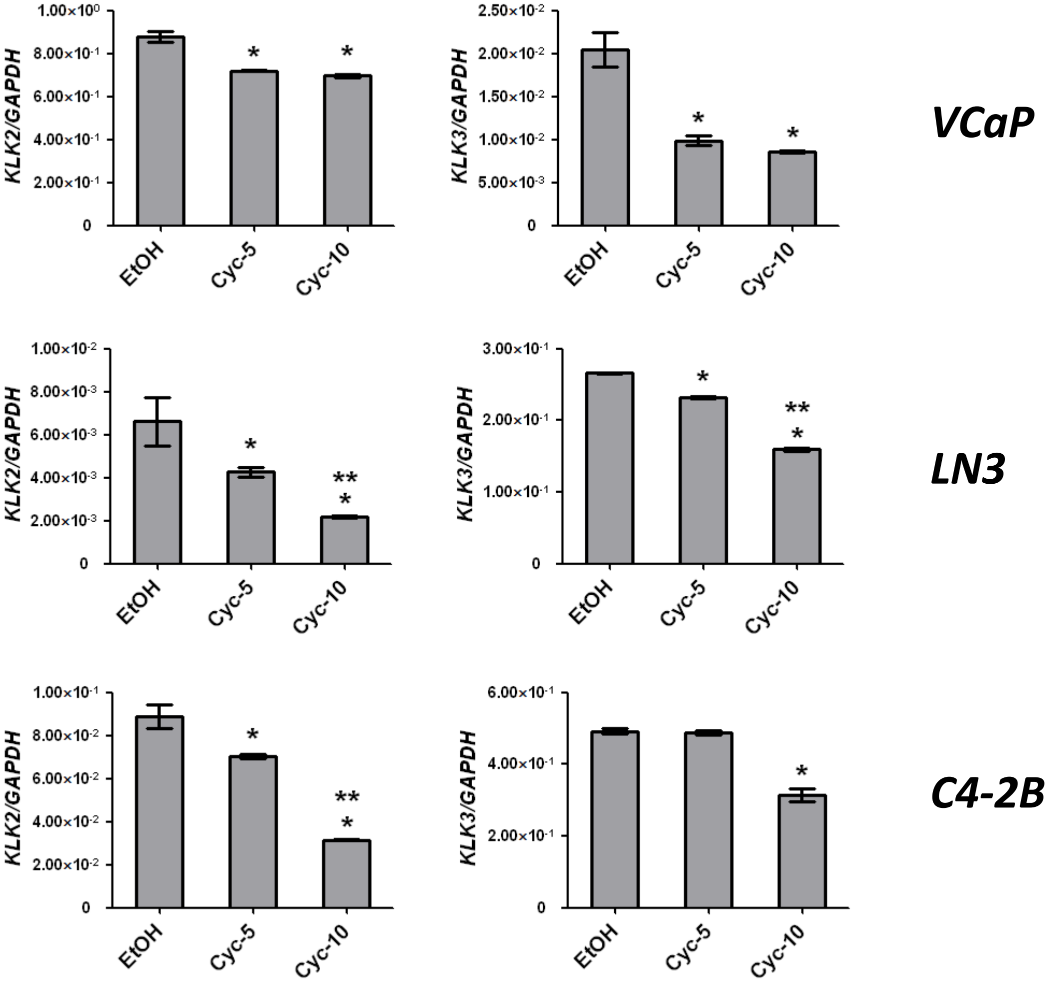
KLK2/GAPDH
KLK3/GAPDH
EtOH
Cyc-5
Cyc-10
VCaP
KLK2/GAPDH
KLK3/GAPDH
EtOH
Cyc-5
Cyc-10
LN3
KLK2/GAPDH
KLK3/GAPDH
EtOH
Cyc-5
Cyc-10
C4-2B

Figure S3

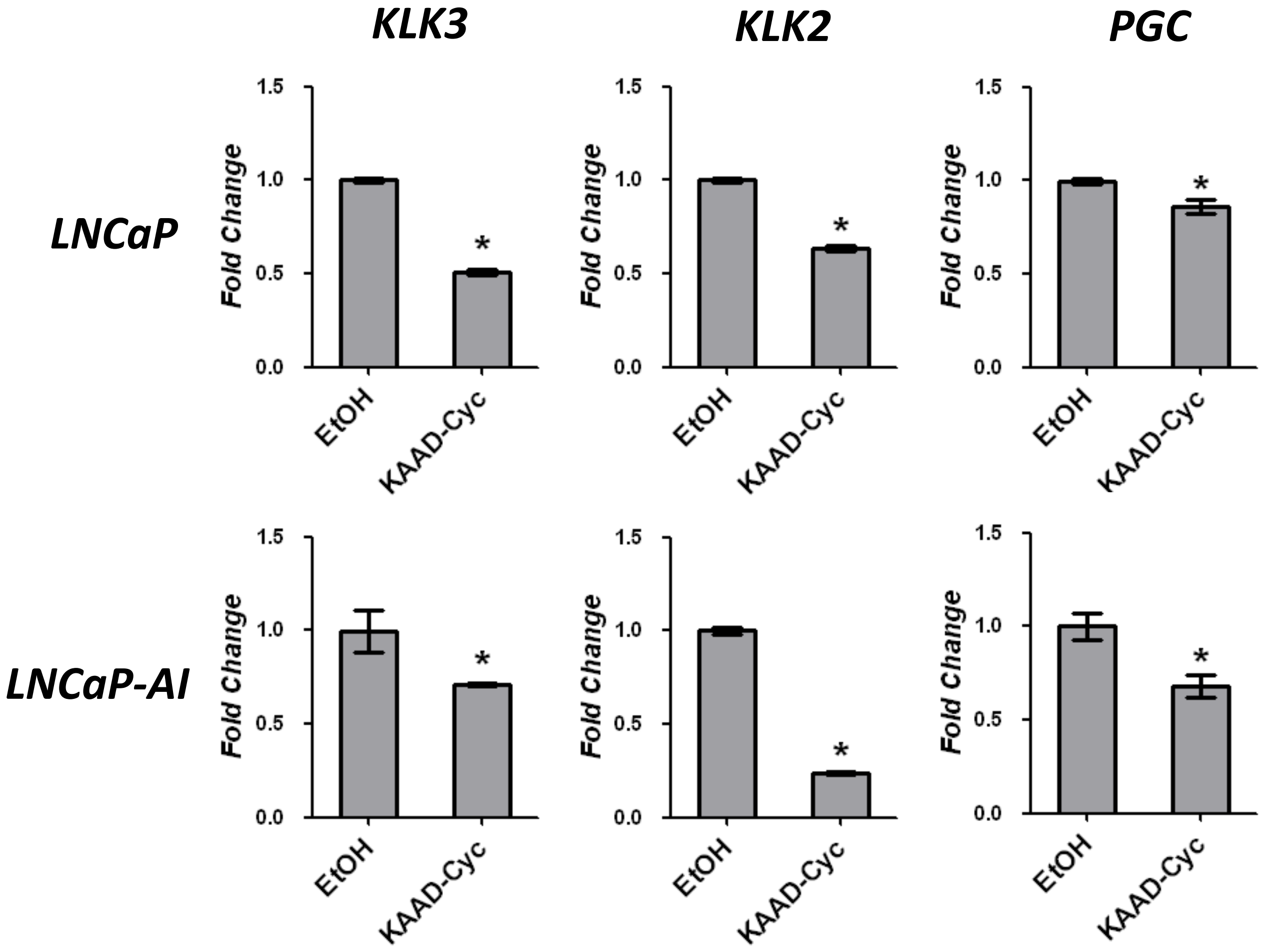

KLK3
KLK2
PGC
LNCaP
LNCaP-AI
Fold Change
1.5
1.0
0.5
0.0
*
EtOH
KAAD-Cyc
