# Supplementary material for: Hedgehog/Gli supports androgen signaling in androgen deprived and androgen independent prostate cancer cells
Source: Mol Cancer. 2010 Apr 26;9:89. doi: 10.1186/1476-4598-9-89 (PMC2873440; doi:10.1186/1476-4598-9-89)
Supplement: Additional file 2 — Supplemental Tables S1-S2; List of PCR primer sets used in experiments and real-time data for Figure1. [file 1476-4598-9-89-S2.PDF]

Table S1. ***Sequences of gene-specific primers used for quantitative Real-Time PCR.***

GAPDH	5'- CCATCACCATCTTCCAGGAGCG -3'
	5'- AGAGATGATGACCCTTTTGGC -3'
AR	5'-GCAGGAAGCAGTATCCGAAG-3'
	5'-CGTTGTCAGAAATGGTCGAA-3'
KLK2	5'-GCTGCCCATTGCCTAAAGAAG-3'
	5'-TGGGAAGCTGTGGCTGACA-3'
KLK3	5'-AGCTGTGGCTGACCTGAAAT-3'
	5'-GTCCTCACAGCTGCCCAC -3'
PGC	5'- GTCCACCTACTCCACCAATG -3'
	5'- TCACTCAAGCCGAACTCCTG -3'
SHH	5'- CCAAAGCGTTCAACTTGTCC -3'
	5'- TTTAAGGAACTCACCCCCAA -3'
SMO	5'- GTCATTCTCACACTTGGGCA-3'
	5'- AAGCTCGTGCTCTGGTCG -3'
GLI1	5'- GGCTCGCCATAGCTACTGAT -3'
	5'- CCAGCGCCCAGACAGAG -3'
GLI2	5'- AGCAGCAGCAGCAACTGTC -3'
	5'- GAATGGCGACAGGGTTGAC -3'
PTCH1	5'- TCTCCAATCTTCTGGCGAGT -3'
	5'- TGGGATTAAAAGCAGCGAAC -3'

Table S2. ***Expression of androgen-regulated genes evaluated by Real-time PCR.*** Values are means ± SEM. Experimental groups are as indicated: LNCaP and LNCaP-AI cells grown under androgen-supplemented (+R1881) or androgen-depleted (-R1881) conditions were treated with cyclopamine (Cyc) or ethanol vehicle control (EtOH) for 3 days before they were extracted for mRNA expression analysis. a Statistical difference ($p<0.05$) between the cyclopamine-treated group (Cyc) and vehicle (ethanol) control group (EtOH). b Statistical difference ($p<0.05$) between the high-dosage cyclopamine (10µM) and low-dosage cyclopamine (5µM) treated groups.

**Experiment Group**		**Gene**	**Expression Level Normalized to GAPDH**	**Fold Change**	**Gene**	**Expression Level Normalized to GAPDH**	**Fold Change**
LNCaP (+R1881)	*EtOH*	KLK3	$1.32 \pm 0.03 \times 10^{-1}$	1.00	KLK2	$1.95 \pm 0.04 \times 10^{-2}$	1.00
	Cyc-5 µM	KLK3	$1.22 \pm 0.04 \times 10^{-1}$	0.93	KLK2	$2.19 \pm 0.11 \times 10^{-2}$	1.12
	Cyc-10 µM	KLK3	$1.14 \pm 0.04 \times 10^{-1}$	0.86	KLK2	$2.10 \pm 0.10 \times 10^{-2}$	1.08
LNCaP (-R1881)	*EtOH*	KLK3	$1.87 \pm 0.22 \times 10^{-2}$	1.00	KLK2	$2.25 \pm 0.08 \times 10^{-3}$	1.00
	Cyc-5 µM	KLK3	$9.12 \pm 0.74 \times 10^{-3}$	0.49 [a]	KLK2	$1.18 \pm 0.04 \times 10^{-3}$	0.52 [a]
	Cyc-10 µM	KLK3	$6.39 \pm 0.33 \times 10^{-3}$	0.34 [a,b]	KLK2	$1.06 \pm 0.02 \times 10^{-3}$	0.47 [a,b]
LNCaP-AI (-R1881)	*EtOH*	KLK3	$7.36 \pm 0.28 \times 10^{-6}$	1.00	KLK2	$1.75 \pm 0.15 \times 10^{-5}$	1.00
	Cyc-5 µM	KLK3	$5.76 \pm 0.57 \times 10^{-6}$	0.78 [a]	KLK2	$1.22 \pm 0.09 \times 10^{-5}$	0.70 [a]
	Cyc-10 µM	KLK3	$3.60 \pm 0.55 \times 10^{-6}$	0.52 [a,b]	KLK2	$9.26 \pm 0.80 \times 10^{-6}$	0.57 [a,b]
LNCaP (+R1881)	*EtOH*	PGC	$1.14 \pm 0.04 \times 10^{-4}$	1.00	SHH	$4.05 \pm 0.63 \times 10^{-7}$	1.00
	Cyc-5 µM	PGC	$1.18 \pm 0.09 \times 10^{-4}$	1.03	SHH	$4.26 \pm 0.63 \times 10^{-7}$	1.05
	Cyc-10 µM	PGC	$1.21 \pm 0.07 \times 10^{-4}$	1.06	SHH	$4.71 \pm 1.05 \times 10^{-7}$	1.16
LNCaP (-R1881)	*EtOH*	PGC	$5.38 \pm 0.63 \times 10^{-5}$	1.00	SHH	$6.71 \pm 1.15 \times 10^{-7}$	1.00
	Cyc-5 µM	PGC	$3.80 \pm 0.14 \times 10^{-5}$	0.71 [a]	SHH	$1.48 \pm 0.16 \times 10^{-6}$	2.21 [a]
	Cyc-10 µM	PGC	$4.00 \pm 0.05 \times 10^{-5}$	0.74 [a]	SHH	$1.76 \pm 0.17 \times 10^{-6}$	2.40 [a]
LNCaP-AI (-R1881)	*EtOH*	PGC	$7.07 \pm 0.94 \times 10^{-6}$	1.00	SHH	$1.26 \pm 0.03 \times 10^{-4}$	1.00
	Cyc-5 µM	PGC	$3.59 \pm 0.67 \times 10^{-6}$	0.51 [a]	SHH	$1.63 \pm 0.09 \times 10^{-4}$	1.30 [a]
	Cyc-10 µM	PGC	$3.63 \pm 0.68 \times 10^{-6}$	0.51 [a]	SHH	$1.51 \pm 0.09 \times 10^{-4}$	1.14 [a]
